# A Multipath Fusion Strategy Based Single Shot Detector †

**DOI:** 10.3390/s21041360

**Published:** 2021-02-15

**Authors:** Shuyi Qu, Kaizhu Huang, Amir Hussain, Yannis Goulermas

**Affiliations:** 1 Department of Computer Science, University of Liverpool, Liverpool L69 7ZX, UK; Shuyi.Qu@xjtlu.edu.cn (S.Q.); J.Y.Goulermas@liverpool.ac.uk (Y.G.); 2 School of Advanced Technology, Xi’an Jiaotong-Liverpool University, Suzhou 215123, China; 3 School of Computing, Edinburgh Napier University, Edinburgh EH11 4BN, UK; a.hussain@napier.ac.uk

**Keywords:** object detection, single shot detector, feature fusion

## Abstract

Object detection has wide applications in intelligent systems and sensor applications. Compared with two stage detectors, recent one stage counterparts are capable of running more efficiently with comparable accuracy, which satisfy the requirement of real-time processing. To further improve the accuracy of one stage single shot detector (SSD), we propose a novel Multi-Path fusion Single Shot Detector (MPSSD). Different from other feature fusion methods, we exploit the connection among different scale representations in a pyramid manner. We propose feature fusion module to generate new feature pyramids based on multiscale features in SSD, and these pyramids are sent to our pyramid aggregation module for generating final features. These enhanced features have both localization and semantics information, thus improving the detection performance with little computation cost. A series of experiments on three benchmark datasets PASCAL VOC2007, VOC2012, and MS COCO demonstrate that our approach outperforms many state-of-the-art detectors both qualitatively and quantitatively. In particular, for input images with size 512 × 512, our method attains mean Average Precision (mAP) of 81.8% on VOC2007 test, 80.3% on VOC2012 test, and 33.1% mAP on COCO test-dev 2015.

## 1. Introduction

Object detection has been a fundamental task in computer vision which aims at localizing objects via bounding boxes and assigning a certain class to each of them. It has been widely adopted on intelligent systems as a crucial component and applied on specific purposes, such as pedestrian detection, face detection, and text detection [1]. Recent deep learning based detectors [2,3,4,5,6,7] have seen great achievements compared with traditional methods. Inspired by these works, we propose a novel efficient yet accurate deep detector. We extend previous work [8] substantially by presenting comprehensive related work, performing a series of additional experiments with more ablation studies, comments on inference time, and failure cases analysis which better show the effectiveness of our method.

Most state-of-the-art object detectors fall into two categories, one-stage detectors and two-stage ones. Two-stage methods are proposed earlier, especially for Faster R-CNN (Regions with CNN features) [4] and R-FCN (Regions with Fully Convolutional Networks) [7]. On the first stage, regions with high probability that contain foregrounds are extracted as proposals. On the second stage, these proposals are sent to a network for classification and bounding-box regression. While archiving high accuracy on benchmark datasets, such as PASCAL VOC [9] and MS COCO [10], two-stage approaches usually run slowly because of the high computation cost of the region proposal generation. Another one-stage branch (e.g., Single Shot Detector (SSD) [6], You Only Look Once (YOLO) [5]) employs proposal-free pipeline, thus it needs few computation resources. These detectors usually adopt fully convolutional architecture and calculate class confidence and regression results directly on predefined boxes. One-stage detectors could run efficiently with accuracy slightly inferior to two-stage ones, thus they are favorable for sensors system due to the crucial need of real-time inference.

The one-stage approach SSD detects objects directly from multiscale features. This feature pyramid consists of last layers of backbone and adjacent convolutional layers. Shallow layers within this pyramid are used to detect small scale objects and deep ones responsible for larger objects. Although efficient, such scheme behaves badly on small objects. Since shallow layers always learn localization information while deep layers have more semantics information [11]. The semantics are crucial for detecting small objects, thus exploiting shallow layers alone is not enough for small scales detection.

To address this issue, some recent works [12,13] introduce a top-down pyramid structure. In order to pass semantics information to shallow layers, they upsample deeper layers before combining them with lower ones. Although borrowing some semantics from deep layers, there is only one feature pyramid engaged. We believe one single pyramid is still not informative enough for accurate detection. Thus, we propose a multipath model which consists of several feature pyramids in order to learn the most informative representations. Although novel in deep object detector, the strategy of multipath is widely applied in computer vision areas [14,15]. Inspired by Feature Fusion Single Shot Multibox Detector (FSSD) [16], we use Feature Fusion Module (FFM) to obtain fused features from base pyramid and obtain our multipath feature pyramids. Then we generate our final features by sending these pyramid features to our Pyramid Aggregation Module (PAM). At last, these informative multiscale features are fed into detection heads for final processing. We conduct extensive experiments on challenging datasets PASCAL VOC and MS COCO, and the results show that our algorithm is better than most stage-of-the-art one-stage object detectors. Below are our main contributions:

- We propose a multipath fusion strategy to enhance the feature pyramid in single shot detector;
- Our feature fusion module and pyramid aggregation module are introduced that proves able to fuse information from base pyramid and generate our informative pyramid efficiently;
- Extensive experiments on PASCAL VOC 2007 & 2012 and MS COCO 2015 show our proposed Multi-Path fusion Single Shot Detector (MPSSD) can outperform other one-stage detectors consistently.

## 2. Related Work

We present the related work by dividing it into three parts. We will first describe the deep learning based object detector, followed by an introduction of single shot detector branch. Finally, we will discuss the feature enhancement in deep detectors.

### 2.1. Deep Object Detector

Traditional object detection methods always rely on hand-crafted features [17,18,19,20,21]. Histogram of Oriented Gradients (HOG) [18] is a representative feature descriptor which can be calculated on densely uniformed cells. Deformable Part-based Model (DPM) is an improved version of HOG which follows the “divide and conquer” scheme to detect an image on different parts during inference [21]. Traditional methods are limited by the hand-crafted features and less efficient computation resources. Recently, thanks to the great achievements of deep convolutional neural networks on computer vision [22,23,24,25,26,27,28,29], many deep learning based detectors are proposed with superior performance compared with conventional methods. R-CNN [2] introduces the idea of region proposals, which are regions with high probabilities to include objects. They extract these proposals at first and send them to CNN for further prediction. This two-stage scheme becomes popular due to its superiority over conventional hand-crafted methods on both accuracy and speed. Fast R-CNN [3] accelerates the training of R-CNN through a novel multitask loss to train the classification and bounding box regression simultaneously. To alleviate the heavy computation cost of proposals generation process in these algorithms, faster R-CNN [4] designs a learning-based region proposal network (RPN) to generate proposals efficiently. R-FCN [7] adopt fully-convolutional networks, and they propose the position sensitive RoI Pooling (PSRoI) to replace the RoI Pooling in faster R-CNN to further improving the accuracy and efficiency. To solve the extreme foreground-background classes imbalance in training, focal loss [30] as an variant of the standard cross entropy loss is proposed to learn more hard examples during training.

### 2.2. Single Shot Detector

Apart from region proposal based detectors, another branch termed as one stage detectors abandons the procedure of proposals extraction in order to achieve faster inference. Among these methods, YOLO [5] probably presents the first one-stage detector which applies successfully classification and bounding box regression directly on each predefined image grid. The drop of proposal generation results in a very high speed, but the accuracy is relatively low. YOLO’s improved versions [31,32] have focused more on this problem, especially for small objects detection.

Another one-stage detector is called SSD [6] that exploits a multiscale fashion to predict objects with various scales, and this improves the performance of one-stage detector significantly. We adopt SSD as our base model since it satisfies the trade-off between speed and accuracy. At first, adopting VGG16 as backbone, SSD modifies the last fully connected layers into convolutional version. For an input image with size 300 × 300, they extract layers conv4_3 with size 38 × 38 and conv_fc7 19 × 19 from backbone. Then, they add several convolution layers conv8_2, conv9_2, conv10_2 and conv11_2 to extract features with size 10 × 10, 5 × 5, 3 × 3 and 1 × 1 respectively. The extracted features establish the detection pyramid in SSD, and each feature is responsible for detecting a particular scale. The shallow layer, i.e., conv4_3, is to detect relative small objects in the image, while the deep layer, i.e., conv9_2, is used to detect large objects. On each feature, they design a number of defined boxes and each box is assumed to include foregrounds. The adjacent head block of each feature is to predict the offsets and classification confidences for these boxes. Finally, a postprocessing called non-maximum suppression (NMS) is used to refine these predicted results.

### 2.3. Deep Feature Enhancement

The quality of feature representations is important for object detection. In order to improve the accuracy of deep detectors, many recent works study how to build more informative features. Feature Pyramid Network (FPN) [12] adopts the top-down feature pyramid design to add lower level features with higher ones. This straightforward design passes higher level semantics to lower levels, thus improving the detection performance especially on small objects. Deconvolutional Single Shot Detector (DSSD) [13] applies the same strategy into vanilla SSD architecture and archieves better performance, but their deconvolution design would hurt the high-efficient property of the original SSD. HyperNet [33] and ION [34] combine the hierarchical features into one layer before the prediction, aiming for building the most representative feature which enjoys both local and global information. In terms of feature enhancement in one-stage detectors, inspired by FPN, StairNet [35] improves the nearest neighbor upsampling with a newly introduced top-down feature combining module and attains even better results. RFBNet [36] proposes a novel Receptive Field Block (RFB) with groups of dilated convolution design, and this module is able to build high-quality representatives through enlarging the receptive field of input features. Some other SSD based methods try to enhance the base features through fusion of different layers with their novel fusion modules [16,37,38]. In this paper, we propose a new multipath fusion strategy to generate multilevel feature pyramids. We illustrate that our SSD based object detector could outperform many counterparts both qualitatively and quantitatively on three benchmark datasets.

## 3. Methodology

Before introducing our novel architecture and modules, we revisit one-stage detector SSD and its variant FSSD. We elaborate our methodology in detail and then offer interpretations accordingly.

### 3.1. Deep Feature Pyramid

Single shot multibox object detector often extracts features from one backbone network and adds several convolution layers to obtain feature pyramid. We denote the feature of the $l^{th}$ layer is $x_{l}\in R^{H\times W\times C}$ (where *H*, *W*, and *C* refer to height, width, channel respectively). The feature pyramid in SSD for multiscale detection is defined as:

(1) $$X_{Detection}=\left\{x_{k},x_{k+1},...,x_{L}\right\},$$

where $x_{l}$ is used to predict objects within a certain range of scale. Specifically, the shallowest feature $x_{k}$ responsible for detecting small objects within inputs, and larger objects are detected in deeper layers. However, as the feature goes deeper, the more semantics it contains, which could provide global information. Shallow features missing this global guidance would lead to misdetection. Thus, SSD has poor accuracy on relatively small objects.

To resolve this problem, many investigations focus on building more informative representations. For instance, the straightforward way is adding higher level semantics to low level local features [13]. However, this strategy may need much heavy computation owning to the complicated element-wise operations between adjacent features. An efficient method is proposed by FSSD [16] to fuse low level and high level information. They concatenate several features and generate the following pyramid the same as SSD. The whole process can be shown as below:

(2) $$X_{Detection}=\left\{x_{k}^{{}^{\prime }},x_{k+1}^{{}^{\prime }},...,x_{L}^{{}^{\prime }}\right\},$$

and each level feature is computed by:

(3) $$x_{l}^{{}^{\prime }}=F\left(\left\{x_{k},x_{k+1},...,x_{k+n}\right\}\right),$$

where *F* indicates the operation of feature fusion and pyramid generation, and the number of base feature from SSD to be fused is (n+1). We can find in (3) only base features from *k* to k+n are used to generate final feature pyramid, while the rest of the base features with rich semantics are discarded. We argue that these features can also be used to generate final pyramid through a dedicated transformation. Thus, we design a multipath routine to maximize the usage of base features. Firstly, we generate several groups of features on base pyramid through feature fusion modules. Then, we pass these pyramids into our aggregation module for final fusion. The detection results are predicted on the final enhanced pyramid:

(4) $$X_{Detection}=\left\{x_{k}^{{}^{\prime \prime }},x_{k+1}^{{}^{\prime \prime }},...,x_{L}^{{}^{\prime \prime }}\right\},$$

and each feature is expressed by:

(5) $$\begin{array}{c} x_{L}^{{}^{\prime \prime }}=A(\left\{F_{1}\left(\left\{x_{k},x_{k+1},...,x_{k+1+n}\right\}\right)\right\},...,\\ \{F_{M}\left(\left\{x_{k+M-1},x_{k+M},...,x_{k+M+n}\right\}\right)\}), \end{array}$$

where *M* is the number of pyramid paths. We apply several feature fusion modules, which is represented by $F_{m}$ and pyramid aggregation module indicated by *A*. More details about these two modules can be found in Section 3.3 and Section 3.4.

The generated final feature pyramid is rich in both localization and semantic information at each level. For instance, the last layer of this pyramid is fused from same size features in each path, and these features are enhanced through nonlinear transformations of different level base features. Our fusion strategy could generate features rich in local and global clues and it is lightweight to be inserted in vanilla SSD model.

### 3.2. MPSSD Architecture

**Figure 1:**
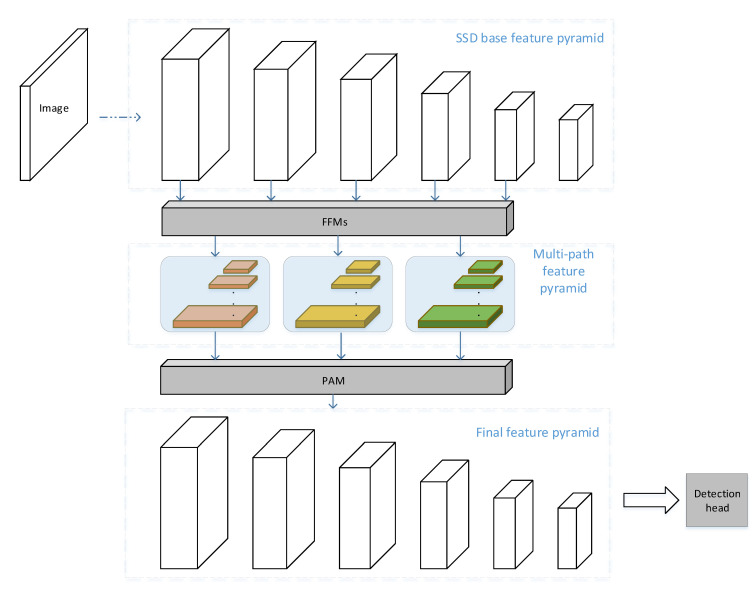
Our model pipeline. From top to bottom, each part indicates: Single Shot Detector (SSD) base feature pyramid, multipath feature pyramids generated through Feature Fusion Modules, final feature pyramid out from pyramid aggregation module.

As shown in Figure 1, our architecture is based on single-stage design in vanilla SSD. The backbone network we use is VGG16. Same as that in SSD, we replace last fc6 and fc7 layers with convolution layers for a fully convolutional fashion. Other base features are generated from the following several convolution layers. These base features are then sent to two modules for enhancement. The enhanced feature pyramid with both local and global information is connected with detection heads for prediction. We illustrate the modules in the following sections.

### 3.3. Feature Fusion Module

**Figure 2:**
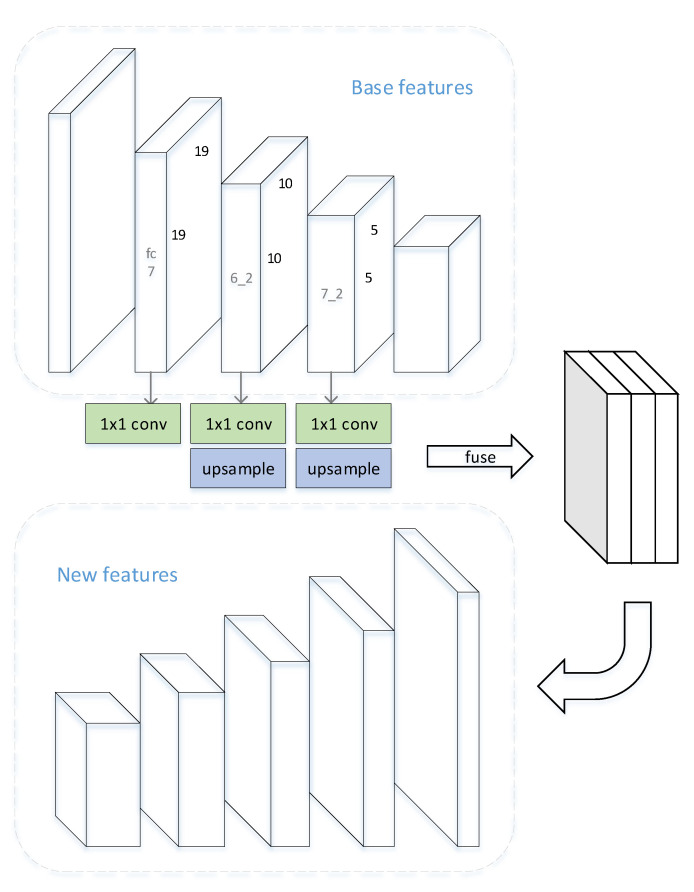
Design of feature fusion module. Top are base features. Three features are selected to be fused. These features are computed through 1 × 1 conv and upsampled into same scale and channel. Following by concatenation to form the aggregation feature. Bottom shows the generated new features from the aggregation.

In order to better integrate various features with different scales, FSSD engages an efficient fusion method. Inspired by the success, we also exploit this module so as to generate our novel multipath feature pyramids. The structure is shown in Figure 2. For an input image of size 300 × 300, we choose layers from conv_fc7, conv6_2 and conv7_2 to fuse. Since these features are in different sizes (size of conv_fc7 is 19 × 19, conv6_2 is 10 × 10, conv7_2 is 5 × 5), smaller features conv6_2 and conv7_2 are upsampled to the same size of conv_fc7 before fusion. We also utilize 1 × 1 convolution before upsampling to reduce the channels into a particular number, and different channel numbers are set in our FFMs. Next, we concatenate these same size features in channel dimension. The feature pyramid is then generate through several 3 × 3 conv + Rectified Linear Unit (ReLU) layers. For multipath pyramids, we repeat this process several times.

### 3.4. Pyramid Aggregation Module

We devise a novel pyramid aggregation module to transform multipath pyramid from feature fusion modules into final detection pyramid. This module is illustrated in the bottom of Figure 1.

The whole process can be divided into two steps. Firstly, we aim to arrange these features into a single pyramid for further detection, thus same scale features are concatenated along the channel dimension. It is noticeable that each path pyramid has a different number of features. On the second step, we adopt attention block in Squeeze-and-Excitation Networks (SENet) [39] to enhance the concatenated features. This attention block is implemented through squeeze and excitation steps. We show its procedure in the following. Firstly on squeeze step, the global average pooling is used to generate $z\in R^{C}$ for input $X\in R^{H\times W\times C}$:

(6) $$z_{c}=\frac{1}{H\times W}\sum_{i=1}^{H}\sum_{j=1}^{W}X_{c}\left(i,j\right).$$

Then, the excitation step generates the attention activation feature *s* by

(7) $$s=F_{ex}\left(z,W\right)=\sigma \left(W_{2}\delta \left(W_{1}z\right)\right),$$

where σ and δ refer to ReLU and sigmoid operation respectively, $W_{1}\in R^{C^{{}^{\prime }}\times C}$ and $W_{2}\in R^{C\times C^{{}^{\prime }}}$, we adopt $C^{{}^{\prime }}=\frac{1}{16}C$ in our experiments. The last stage outputs the result $\tilde{X}\in R^{H\times W\times C}$ by

(8) $${\tilde{X}}_{c}=F_{scale}\left(X,s_{c}\right)=s_{c}\cdot X_{c}.$$

## 4. Results and Discussion

We conduct extensive experiments on three benchmarks PASCAL VOC2007, PASCAL VOC2012, and MS COCO. As for the evaluation metrics, in VOC, predicted boxes that have Intersection over Union (IoU) with the ground truth higher than 0.5 are defined as positive results. In COCO, the metrics are split into different parts based on different Intersection over Union (IoU) settings. Our implementation is based on Pytorch 0.4.0. Backbone network VGG16 is pretrained on ImageNet [40] with modification into fully convolutional version. As for the training settings, we keep them mostly the same as the settings in SSD for fair comparison. On the other hand, we adopt the network initial method from [41].

### 4.1. Dataset

**Figure 3:**
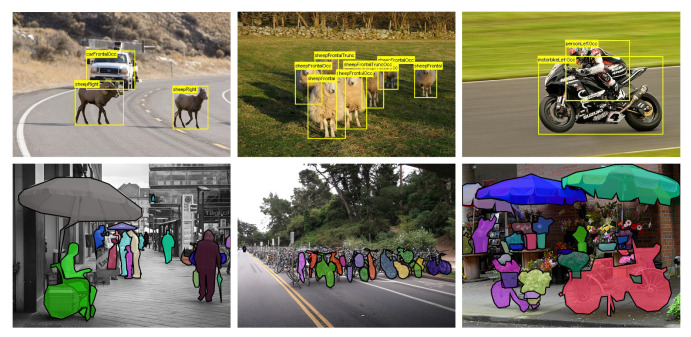
Some object detection examples in Pascal VOC (**upper**) and MS COCO (**lower**).

We applied two popular datasets, PASCAL VOC and MS COCO, during training and testing. The PASCAL VOC dataset contains images collected from the Internet. The 2007 and 2012 versions are two most used in object detection. They are categorized into 20 classes since 2007 based on four groups: person, animal, vehicle, and indoor. MS COCO is a newly established dataset for object detection, segmentation, and captioning. Compared with VOC, MS COCO adopts annotations not only for the bounding box, but for each object it is labeled with instance segmentation for more precise localization. This dataset is more challenging and with more than 200,000 images categorized into 80 classes, and there are more labeled objects per image compared with PASCAL VOC. Table 1 shows the detailed statistics of these datasets. For intuitive observations, we also show some examples from these two datasets in Figure 3.

### 4.2. PASCAL VOC 2007

All the experiments on PASCAL VOC apply the popular split, which uses the union of VOC2007 trainval and VOC2012 trainval as the training data, and VOC2007 test which contains 4952 images for testing. For training on inputs with scale 300×300, the batch size is set at 24 on single NVIDIA 1080Ti GPU. The initial learning rate is $4\times 10^{-3}$. The learning rate decreases to $4\times 10^{-4}$ at 150 epoch and to $4\times 10^{-5}$ at 200 epoch. We finish our training on 250 epoch.

Table 2 shows the results on VOC2007. Our model outperforms vanilla SSD significantly. As for 300 × 300 inputs, by embedding our algorithm into SSD, the mAP (mean Average Precision) improves from 77.5% to 80.3%, while increases from 79.5% to 81.8% on 512 × 512 inputs. We also archive better performance compared with DSSD with ResNet101 [22] as backbone. We find FSSD with similar fusion strategy but with less features is inferior to our method. Based on the mAP, we archive 1.5% higher on 300 × 300 and 0.9% higher on 512 × 512, and this show our multipath design is effective. Compared with top-down design, such as StairNet, the 1.5 point higher accuracy proves our architecture helps to learn more powerful features. Our method also reaches better performance compared with these two-stage algorithms.

### 4.3. PASCAL VOC 2012

We adopt the same training setting as that in our VOC2007 experiments to evaluate our model on dataset VOC2012 test, which contains 10,991 images in total. From Table 3, our method achieved the same results as the evaluation on VOC2007 dataset. For both input resolutions, our model improves significantly from the SSD baseline. Compared with other single-stage methods, our MPSSD reached the best performance under the same training settings. It is noteworthy that FSSD obtained better results via adopting additional training dataset (MS COCO). These results again show the advantages of our proposed method.

### 4.4. MS COCO

We also conduct experiments on a more challenging dataset, MS COCO, for further evaluation. For the training and validation split, we adopt trainval35k for training, minival for validation, and test-dev 2015 for testing. For input on 300 × 300, our batch size is 16 on single NVIDIA 1080Ti GPU. The learning rate is initialized at at $2\times 10^{-3}$ with schedule decay on epoch 90 and 120 by the factor of 10. The training ends at 150 epoch.

Shown in Table 4, the evaluation metrics are acquired through official server on CodaLab. For 300 × 300 input images, our model outperforms SSD in a large margin, mAP improves from 25.1% to 27.5%. Compared with FSSD, our model obtains 0.4% higher accuracy, which shows our effectiveness. For 512 × 512 input images, our model archives 33.1% accuracy, which is 4.3% and 1.3% higher than SSD and FSSD, respectively. Our method is only 0.1% inferior to DSSD513, and we believe this mainly because they adopt a more powerful backbone ResNet101. Specifically, we can find ours works best on small scale detection among these algorithms.

### 4.5. Inference Time Analysis

We take into consideration the inference time of our algorithms because of the real-time requirement of sensor system. To compare fairly, for 4952 images in VOC 2007 test with size 300 × 300, we average their total inference time without considering the postprocessing (NMS) step. All the results are evaluated on one NVIDIA 1080Ti GPU with batch size set at 1, and these methods are trained under the same settings.

**Figure 4:**
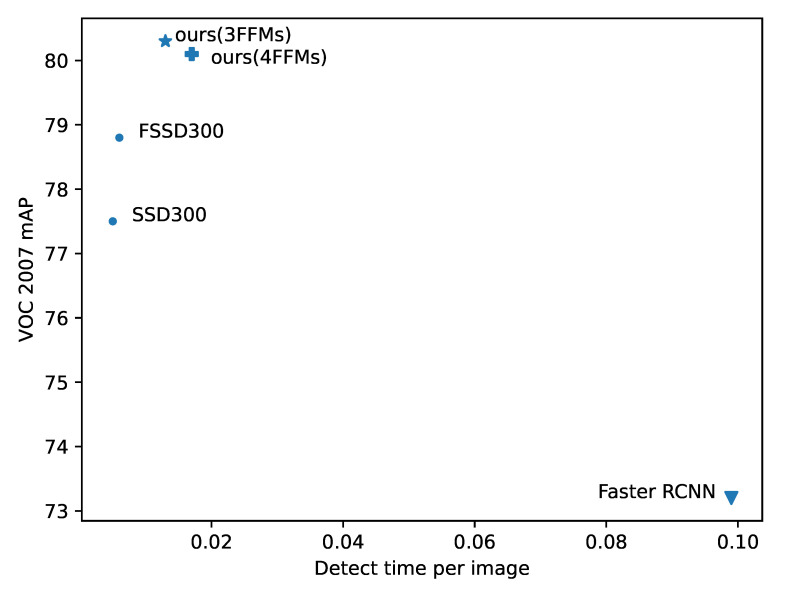
Comparison on detect time of each image. In the figure, 3FFMs indicates the model which adopts 3 feature fusion modules, while 4FFMs applies 4 feature fusion modules.

We analyze how our newly introduced modules affect the inference speed. The results are shown in Figure 4. Compared with vanilla SSD, our methods could reach a much higher accuracy with little sacrifice of inference time. In addition, in contrast to FSSD, our multiple design of FFMs and Pyramid Aggregation Module (PAM) would not spend too much computation with better performance. We also compared one-stage baseline Faster RCNN, and our method could again achieve higher accuracy with much less time cost. Since other one-stage methods are based on Faster RCNN baseline, we do not make the comparisons for simplicity. Finally, we test how the number of Feature Fusion Module (FFM) affects the detect time. From Figure 4, we find that one more FFM could reduce the inference speed a little, and thus it is essential to select the optimal parameters.

### 4.6. Qualitative Evaluation

**Figure 5:**
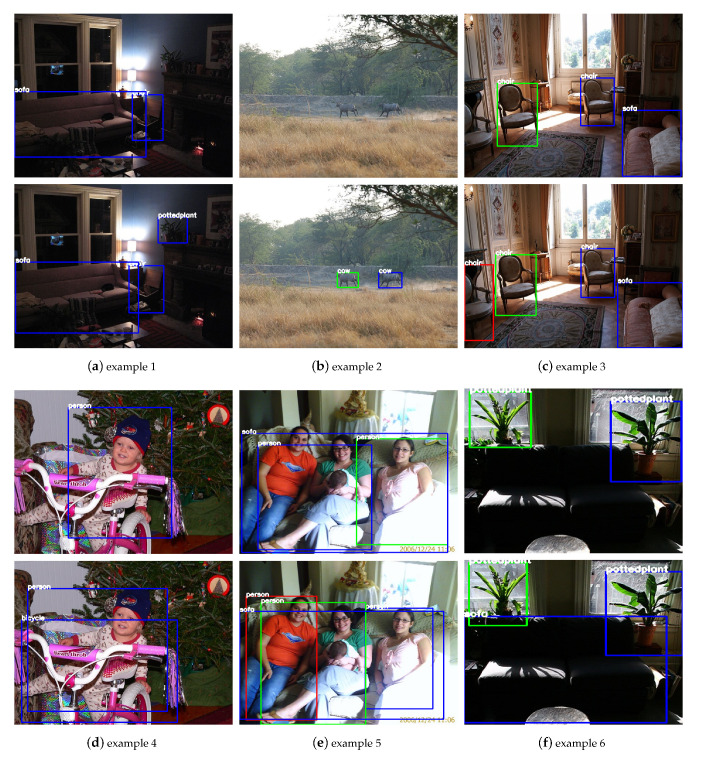
Some detection results from VOC 2007 test. Upper: SSD. Lower: Ours. See more details in Section 4.6. Adapted from [8].

In this part, we show some qualitative results on VOC 2007 test. Seen from each example in Figure 5, the result of SSD is in the upper row and ours is in the lower row. Boxes with classification confidences higher than 0.6 are chosen and each category is labeled on the top of box.

For the first example, the potted plant with low illumination is misdetected in SSD, while our model could handle this illumination variance and detect it successfully. The second example shows our model works better on detecting small objects, such as cows with this image. The following three examples indicate our model outperforms baseline on occlusion objects detection. For the last example, the sofa is neglected by SSD, while it is detected by our model. This proves our learned semantics help to infer objects under certain environment.

### 4.7. Ablation Study on Feature Fusion Modules

To show the effectiveness of our module, we conduct experiments on different settings of feature fusion modules. We conduct all the experiments on VOC 2007 test with 300 × 300 inputs but we should bear in mind that a similar conclusion can also be made in the other datasets.

We run experiments with different numbers of FFMs and different choices of channel numbers. As seen from Table 5, the results on the first column come from vanilla SSD. Through adding FFMs, we could find the accuracy improves a lot progressively. The model with three FFMs and with channel equals to 128 on third FFM reaches the highest accuracy, and we choose this as our final model.

### 4.8. Failure Cases Analysis

**Figure 6:**
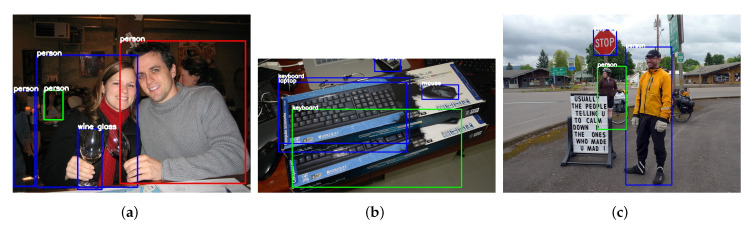
Some failure cases. The results (**a**–**c**) are all from MS COCO 2015 test.

In this part, we analyze the shortcomings of our method. Figure 6 shows some examples with misdetection or incorrect classification from COCO 2015 test. In Figure 6a, the wine glass held by the man is not detected, and we conjecture that the failure is due to the object being tilted with a certain degree, which is not the same as its normal state. On the contrary, the wine glass held by the woman vertically is well-recognized. This case has shown that our model is unable to detect objects with abnormal positions. In Figure 6b, the top keyboard is classified into laptop incorrectly. Although the object “laptop” consists of “keyboard” and “screen”, this incorrect classification is probably because the model is incapable of learning the distinguishable features of these two objects. Finally, in Figure 6c, the green signpost behind the man with yellow coat is misdetected. Since this object is in abnormal aspect ratio (tall and slender), this kind of shape is hard to be recognized mainly because of the weakness of anchor design scheme. We adopt vanilla SSD anchor design which may not be able to cover objects with arbitrary shape, especially for objects with large aspect ratio.

### 4.9. Future Work

We consider three aspects for the future improvements. First, to improve the accuracy of our current model, we believe further optimizing of feature pyramid could reach a more accurate detection. Precisely speaking, the current method builds a fixed number of pyramids, which may only optimize on low resolution inputs, i.e., 300 × 300. With rapid improvements on camera devices, high resolution detection is highly required and thus we need to redesign an optimal strategy. Second, to speed up the inference time of our model, we could focus on alleviating the tedious computation on detection head. The predictions on defined boxes are redundant due to the original design in SSD, and this hurts the speed a great deal. Finally, we will extend the proposed model in various other domains of intelligent systems and sensor applications. As a one-shot detector both efficiently and accurately, the proposed multipath fusion strategy based algorithm could find its applications extensively.

## 5. Conclusions

We devise a novel SSD based one stage detector. Different from prior methods, we propose a multipath fusion strategy to fully utilize the local and global information from different layers. In order to aggregate this information, we first use feature fusion modules to generate feature pyramids, followed by pyramid aggregation module to fuse and enhance relative features. The generated pyramid is suitable for detection in multiscale manner with rich information. Quantitative and qualitative experiments on PASCAL VOC and MS COCO show our MPSSD outperforms vanilla SSD significantly. While for other single stage counterparts, our method archives comparable results without hurting efficiency.

## Figures and Tables

**Table 1:** Dataset statistics of PASCAL Visual Object Classes (VOC) & Microsoft Common Objects in COntext (MS COCO).

Name	Train Images	Validation Images	Test Images	Category	Total Annotated Objects
VOC 2007 [9]	2501	2510	4952	20	9963
VOC 2012 [9]	5717	5823	10,991	20	11,530
MS COCO 2015 [10]	82,783	41,620	125,436	80	2,500,000

**Table 2:** PASCAL VOC 2007 test results. Faster R-CNN (Regions with CNN features), HyperNet, Inside-Outside Net (ION) and R-FCN (Regions with Fully Convolutional Networks) adopted inputs with minimum size 600, while StariNet kept with 300 × 300. Data from [8].

Method	Backbone	mAP (%)	Aero	Bike	Bird	Boat	Bottle	Bus	Car	Cat	Chair	Cow	Table	Dog	Horse	Mbike	Person
Faster [4]	VGG16	73.2	76.5	79.0	70.9	65.5	52.1	83.1	84.7	86.4	52.0	81.9	65.7	84.8	84.6	77.5	76.7
Faster [4]	ResNet101	76.4	79.8	80.7	76.2	68.3	55.9	85.1	85.3	89.8	56.7	87.8	69.4	88.3	88.9	80.9	78.4
HyperNet [33]	VGG16	76.3	77.4	83.3	75.0	69.1	62.4	83.1	87.4	87.4	57.1	79.8	71.4	85.1	85.1	80.0	79.1
ION [34]	VGG16	76.5	79.2	79.2	77.4	69.8	55.7	85.2	84.2	89.8	57.5	78.5	73.8	87.8	85.9	81.3	75.3
R-FCN [7]	ResNet101	80.5	79.9	87.2	81.5	72.0	69.8	86.8	88.5	89.8	67.0	88.1	74.5	89.8	90.6	79.9	81.2
SSD300 [6]	VGG16	77.5	79.5	83.9	76.0	69.6	50.5	87.0	85.7	88.1	60.3	81.5	77.0	86.1	87.5	83.9	79.4
DSSD321 [13]	ResNet101	78.6	81.9	84.9	80.5	68.4	53.9	85.6	86.2	88.9	61.1	83.5	78.7	86.7	88.7	86.7	79.7
StairNet [35]	VGG16	78.8	81.3	85.4	77.8	72.1	59.2	86.4	86.8	87.5	62.7	85.7	76.0	84.1	88.4	86.1	78.8
FSSD300 [16]	VGG16	78.8	-	-	-	-	-	-	-	-	-	-	-	-	-	-	-
Ours300	VGG16	**80.3**	83.5	86.7	79.0	74.4	59.9	87.4	87.4	86.5	64.9	88.1	77.9	86.6	88.0	87.2	80.9
SSD512 [6]	VGG16	79.5	84.8	85.1	81.5	73.0	57.8	87.8	88.3	87.4	63.5	85.4	73.2	86.2	86.7	83.9	82.5
DSSD513 [13]	ResNet101	80.0	92.1	86.6	80.3	68.7	58.2	84.3	85.0	94.6	63.3	85.9	65.6	93.0	88.5	87.8	86.4
FSSD512 [16]	VGG16	80.9	-	-	-	-	-	-	-	-	-	-	-	-	-	-	-
Ours512	VGG16	**81.8**	87.9	88.4	81.7	76.7	65.1	88.5	88.8	87.9	66.9	89.0	76.9	86.1	89.9	87.4	84.6

**Table 3:** PASCAL VOC 2012 test results from official evaluation server. For training data: “07+12”: union of VOC2007 trainval and VOC2012 trainval; “07++12”: union of VOC2007 trainval and test and VOC2012 trainval; “07+12+S” union of VOC2007 trainval and VOC2012 trainval, plus SBD segmentation labels [42]; “07++12+COCO”: union of “07++12” and COCO trainval35k.

Method	Backbone	Data	mAP (%)
Faster [4]	VGG16	07++12	70.4
Faster [4]	ResNet101	07++12	73.8
HyperNet [33]	VGG16	07++12	71.4
ION [34]	VGG16	07+12+S	76.4
R-FCN [7]	ResNet101	07++12	77.6
SSD300 [6]	VGG16	07++12	72.4
DSSD321 [13]	ResNet101	07++12	76.3
StairNet [35]	VGG16	07++12	76.4
FSSD300 [16]	VGG16	07++12++COCO	82.0
Ours300	VGG16	07++12	**77.7**
SSD512 [6]	VGG16	07++12	74.9
DSSD513 [13]	ResNet101	07++12	80.0
FSSD512 [16]	VGG16	07++12++COCO	84.2
Ours512	VGG16	07++12	**80.3**

**Table 4:** MS COCO test-dev2015 results. Note: The “+++” includes box refinement, context, and multiscale testing. Data from [8].

Method	Backbone	Data Split	Avg. Precision, IoU:			Avg. Precision, Area		
			0.5:0.95	0.5	0.75	S	M	L
Faster [4]	VGG16	trainval	21.9	42.7	-	-	-	-
Faster+++ [22]	ResNet101	trainval	34.9	55.7	-	-	-	-
ION [34]	VGG16	train	23.6	43.2	23.6	6.4	24.1	38.3
R-FCN [7]	ResNet101	trainval	29.2	51.5	-	10.3	32.4	43.3
SSD300 [6]	VGG16	trainval35k	25.1	43.1	25.8	6.6	25.9	41.4
DSSD321 [13]	ResNet101	trainval35k	28.0	46.1	29.2	7.4	28.1	47.6
FSSD300 [16]	VGG16	trainval35k	27.1	47.7	27.8	8.7	29.2	42.2
Ours300	VGG16	trainval35k	27.5	47.8	28.5	10.2	30.2	41.0
SSD512 [6]	VGG16	trainval35k	28.8	48.5	30.3	10.9	31.8	43.5
DSSD513 [13]	ResNet101	trainval35k	33.2	53.3	35.2	13.0	35.4	51.1
FSSD512 [16]	VGG16	trainval35k	31.8	52.8	33.5	14.2	35.1	45.0
Ours512	VGG16	trainval35k	33.1	53.8	35.1	17.1	36.0	45.7

**Table 5:** Ablation study on VOC 2007 test. In the table, ffm3_c indicates the channel number of third feature fusion module.

1 FFM		✓				
2 FFMs			✓			
4 FFMs				✓		
3 FFMs with ffm3_c = 256					✓	
**3 FFMs with ffm3_c = 128 (final model)**						✓
mAP(%)	77.5	79.0	79.7	80.1	79.8	**80.3**

## Data Availability

PASCAL VOC: http://host.robots.ox.ac.uk/pascal/VOC/; MS COCO: https://cocodataset.org/.
